# Renal myxosporidiosis by an unknown Bivalvulidan myxozoan parasite in Murray River turtles (*Emydura macquarii)* in Australia

**DOI:** 10.1016/j.ijppaw.2025.101061

**Published:** 2025-03-26

**Authors:** Zachary Low, Telleasha L. Greay, Swaid Abdullah, Phoebe A. Chapman, Viviana Gonzalez-Astudillo

**Affiliations:** a The University of Queensland, School of Veterinary Science, Gatton, Queensland, Australia; b Helicobacter Research Laboratory, The Marshall Centre, The University of Western Australia, Crawley, Western Australia, Australia; c University of Otago, Department of Marine Science, Dunedin, Otago, New Zealand

**Keywords:** Australia, Murray river turtle, Myxozoa, Kidney, Parasite

## Abstract

This case series provides the first published record of a myxozoan parasite in Murray River turtles (*Emydura macquarii*) in Australia. Thirteen turtles were captured for an eco-toxicology study and underwent postmortem examinations. From these, three were found to have interstitial nephritis and spores within the affected renal tubules. Molecular characterisation was performed with PCR which yielded positive results for myxozoan DNA in the three infected samples. DNA sequencing and phylogenetic analysis of 18S rRNA sequences positioned the unknown species in a distinct clade, closely related to, but separate from, histozoic clades II and III. This discovery contributes significantly to the understanding of myxozoan diversity and ecology, highlighting a potential new threat to the health of Murray River turtle populations and possibly other aquatic reptiles. The discovery of this myxozoan species not only broadens the known host range of myxozoans but also raises concerns about the conservation of affected turtle populations due to its possible pathogenic nature.

## Highlights

• Unknown myxozoa were documented within the cortical renal tubules of Australian Murray River turtles (*Emydura macquarii*).
• Infection was associated with variable degrees of chronic inflammation.
• Molecular analysis revealed the unknown myxozoan species belongs to a distinct genetic clade.
• Findings highlight potential conservation concerns for Australian freshwater turtles.

## Graphical abstract

**Figure:**
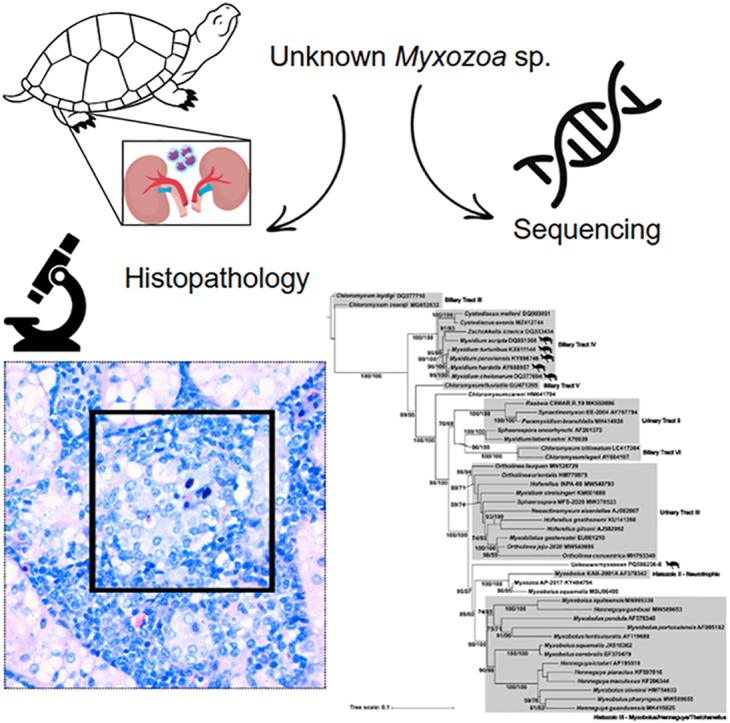

## 1 Introduction

Myxozoans are obligate, metazoan endoparasites that form spores as part of their complex life cycles which can involve a wide range of host species, primarily fish. Myxozoans are considered endoparasitic cnidarians and have been extensively studied in fish populations due to their economic impact in commercially farmed fish (Okamura et al., 2018). However, there are also reports of myxozoan infections in invertebrates, amphibians, birds, mammals and reptiles. Myxozoan infections exhibit a wide range of pathogenic effects, with some causing minimal harm while others lead to severe illness, high mortality, or significant impacts on host reproduction (Sitjà-Bobadilla, 2008). These infections may be coelozoic – located within host cavities – or histozoic – living between host cells or tissues. While myxozoans are typically classified as extracellular parasites, certain stages of their life cycle occur intracellularly, enabling them to evade host immune defences (Holzer et al., 2021). The myxozoan life cycle in turtles likely follows an indirect cycle involving an invertebrate host, such as annelid worms. Actinospores infect turtles via ingestion or skin penetration, developing into myxospores shed through faeces or tissue decomposition. These are then ingested by an invertebrate host, completing the life cycle (Okamura et al., 2018). Myxozoan infections are rarely reported in turtles globally, and no prior molecularly confirmed cases have been documented in Australia. However, there have been unpublished reports of myxozoan infections in Murray River turtles, as well as in Bellinger River snapping turtles (*Myuchelys gorgesi*) and Eastern long-necked turtles (*Chelodina longicollis*). These infections were identified through histological examination, but molecular analysis was not performed to confirm the species (Australian Registry of Wildlife Health, unpublished data). Therefore, this case series represents the first molecularly confirmed instance of myxozoan infection in Murray River turtles in Australia, providing new insights into the diversity and ecological impact of myxozoans in Australian freshwater turtle species.

The Murray River turtle (*Emydura macquarii*) also known as the Eastern Short-necked turtle, is a freshwater turtle native to south-eastern Australia. They inhabit the Murray River Basin and all its major tributaries along with other coastal rivers in Queensland (Qld), New South Wales, Victoria and South Australia. *E. macquarii* is typically a medium-sized turtle species, with a carapace length of up to 40cm. Males are smaller than females and can grow to about 2.2kg in comparison to over 4kg in females. This turtle species is omnivorous, with diets mainly consisting of aquatic plants, insects, crustaceans, molluscs and small fish. According to the International Union of Conservation of Nature (IUCN) Red List, *E. macquarii* is categorised as Least Concern, but has been listed as Vulnerable by the South Australian Department for Environment and Water (SA/DEW) as well as the Victoria Department of Environment, Land, Water and Planning (VIC/DEWLP). Main threats to this species include habitat loss and altered hydrology, compromised water quality due to pollution as well as predation on both young and adults. This case series describes renal myxosporidiosis associated with a new species of myxozoa found in three *E. macquarii,* documenting the first report for this parasite in Australian turtles, and describing its phylogeny and associated renal pathology.

## 2 Materials and methods

### 2.1 Sample collection

A total of 13 wild adult female *E. macquarii* carcasses were acquired secondarily as part of another collaborative project with Department of Environment and Science, Commonwealth Scientific and Industrial Research Organisation (CSIRO), The University of Queensland, and Griffith University (AEC permit SA, 2018/11/663).

For the primary study, live turtles were obtained from four different sites along freshwater reservoirs across Qld during the Australian spring in September 2021. As the previous study was part of an ongoing investigation on the impact of industrial per- and polyfluroalkyl substances (PFAS) pollution on *E. macquarii*, the locations of the sites at which the turtles were captured was confidential and undisclosed. Live turtles were transported in blacked-out tubs to the Veterinary Laboratory Services at the School of Veterinary Science (SVS), The University of Queensland (UQ) (Gatton, Qld). Upon arrival at the laboratory, the turtles were sedated with Zoletil® (tiletamine and zolazepam; 15mg/kg) prior to the performance of a clinical examination. The turtles were subsequently euthanised with Lethabarb® (pentobarbitone sodium; 2.5mL) and subjected to postmortem examination. After postmortem examination, the carcasses were donated to the Veterinary Laboratory Services for use in this study. Thus, while the turtles were originally acquired for the PFAS study, their cadavers were utilised secondarily for the present research.

### 2.2 Gross pathological examination

Systematic postmortem examinations were conducted on all 13 turtles following established internal UQ SVS postmortem protocols. Samples of all major organ systems, including the brain, skin, appendicular skeletal muscle, thymus, major arteries, pharynx, trachea, lungs, heart, oesophagus, stomach, intestines, pancreas, spleen, liver, kidneys and reproductive tracts were collected for histopathology. A representative section from each kidney of each turtle was collected and submitted for histological analysis.

### 2.3 Histological sample processing

Collected tissue samples were fixed in 10 % neutral buffered formalin for 7 days before subsequently processing to produce 4μm-thick, haematoxylin and eosin (H&E)-stained slides following validated laboratory protocols (Fischer et al., 2008).

### 2.4 Ancillary testing

Faecal flotation tests were conducted following established protocols for the identification of parasite stages, ova or oocysts (Ballweber et al., 2014). Histochemistry using Giemsa stain was conducted on kidney samples of one of the 3 turtles infected with the myxozoa following established laboratory protocols (Lester, 2018).

Blood samples were collected from each turtle and submitted to IDEXX (Qld) for reptile biochemistry profile analysis. The biochemistry analysis included the evaluation of glucose, urea, uric acid, phosphorus, calcium, total protein, albumin, globulin, albumin:globulin ratio, aspartate aminotransferase (AST), glutamate dehydrogenase (GLDH), cholesterol, creatinine kinase (CK), haemolysis and lipaemia index as well as bile acids.

### 2.5 Molecular analysis

Samples of all major organs, including kidneys, were collected and frozen at −20 °C. Frozen kidney tissue samples were available from only 10 of 13 turtles, which were submitted for DNA extraction and molecular analysis. DNA extractions was performed using Qiagen DNeasy Blood and Tissue kit (Qiagen, Hilden, Germany) following manufacturer's protocol.

PCR amplification focused on the 18S rDNA region and used a nested PCR approach, with the first round using generic eukaryote primers, and the second round using various myxosporean-specific primers (Table 1). Round 1 PCR products, either undiluted or diluted 1:5 depending on quantity of product (determined by gel visualisation), were used as templates for round 2 reaction. Reactions comprised of 1 μl DNA template, 10 μl MyTaq Reaction Buffer (Bioline, London, UK), 0.2 μl MyTaq DNA Polymerase (Bioline), 0.8 μl of each primer at 10 mM, with the remainder made up of nuclease free water to a total volume of 20 μl. PCR conditions comprised of an initial denaturing step of 94 °C for 2 min, followed by 30 cycles of denaturation at 95 °C for 50 s, annealing for 50 s, and extension at 70 °C for 60 s, followed by a final extension step of 72 °C for 2 min. Annealing temperatures for each primer pair are shown in Table 1. PCR products were visualised on a 1 % agarose gel using TAE buffer stained with SYBR Safe (Invitrogen, Massachusetts, USA) and purified using ExoSAP-IT or a Nucleospin Gel & PCR Cleanup kit (Macherey-Nagel, Düren, GER) prior to submission to the Genetic Analysis Service at the University of Otago, New Zealand for Sanger sequencing.

**Table 1:** Details of primers used in amplifying the partial 18S rRNA of myxozoa. The first round of the nested PCRs used primer pair 1, with the second round using one of the remaining pairs to produce overlapping fragments. Product sizes are approximate.

Round	Pair	Name	Sequence (5’ – 3′)	Annealing temp. (°C)	Product size (bp)	Reference
1	1	18e	TGG TTG ATC CTG CCA GT	63	1683–1930	Hillis and Dixon (1991)
		18g	GGT AGT AGC GAC GGG CGG TGT G			
2	2	Myxgp2f	TGG ATA ACC GTG GGA AA	58	500	Kent et al. (1998)
		ACT1r	AAT TTC ACC TCT CGC TGC CA			(Hallett and Diamant, 2001)
2	3	Myxgen3f	GGA CTA ACR AAT GCG AAG GCA	58	550	Kent et al. (2000)
		Myxgen4r	ACC TGT TAT TGC CAC GCT			
2	4	Myxgp2f	TGG ATA ACC GTG GGA AA	58	900	Kent et al. (1998)
		Myxgen2r	CAR ATG CYT TCG CWY TTG TTA			Kent et al. (2000)
2	5	Myxgp2f	TGG ATA ACC GTG GGA AA	58	1600	Kent et al. (1998)
		Myxgen4r	ACC TGT TAT TGC CAC GCT			Kent et al. (2000)
2	6	Myxo1f	CTG CCC TAT CAA CTW GTT	58	700	Kent et al. (2000)
		Myxgen2r	CAR ATG CYT TCG CWY TTG TTA			

Sequences were inspected and assembled into contigs using Snapgene v7.0.3, which were used for subsequent phylogenetic analysis. A blastn search (Altschul et al., 1990) was completed to verify the sequence identity and to select sequences for phylogenetic analysis. Genbank database sequences were selected based either on similarity scores, or as representatives of major myxozoan clades or other turtle/reptile host species and aligned using Muscle v3.8.1551 (Edgar, 2004). GBlocks 0.91b (Talavera and Castresana, 2007) was used to remove poorly aligned regions, with less stringent settings selected (allowed smaller final blocks, gap positions within final blocks, and less stringent flanking positions). IQTree v2.2.2.3 (Nguyen et al., 2015) was used to infer a maximum likelihood tree; the GTR + F + I + R3 model was selected following analysis by the inbuilt ModelFinder (Kalyaanamoorthy et al., 2017) and 1000 ultrafast bootstrap (UFBoot2 – (Hoang et al., 2018)) replicates were specified. Bayesian inference (MrBayes v3.2.7 (Ronquist et al., 2012)) was used to construct a second tree with model nst = 6, Nucmodel 4x4 and Invgamma as the closest approximation to the GTR + F + I + R3 model. A total of 100,000 generations were run, with sampling occurring every 100 generations. The first 10 % of samples were discarded as burn-in, and convergence confirmed using Tracer v1.7 (Rambaut et al., 2018). Tree figures were constructed using Interactive Tree of Life (version 7) with final formatting completed with Inkscape (version 1.2.0).

## 3 Results

An unknown myxozoan parasite was identified within the lumen of renal tubules in turtles 6, 11, and 13, associated with interstitial or tubulointerstitial lymphoplasmacytic and or histiocytic/granulomatous, and mildly heterophilic inflammation. In Turtles 1, 5, 10, and 12, there was also evidence of renal inflammation, however the parasite was not observed. Tubular degeneration was noticed in turtles 1, 5, 6, 11 and 12. Intra-lesional mineral deposits (Turtle 1, 5) and refractile, unidentified crystals (Turtle 1) affecting the cortical tubular epithelium were also observed.

### 3.1 Case series descriptions

All turtles were clinically normal following a thorough clinical examination and were subsequently euthanised. Body condition was adequate in both myxozoan parasite-infected and non-infected turtles and all the turtles that were infected had shelled oviducal eggs. The description of three turtles infected with myxozoan parasites is provided below.

#### 3.1.1 Turtle 6

**Fig. 1:**
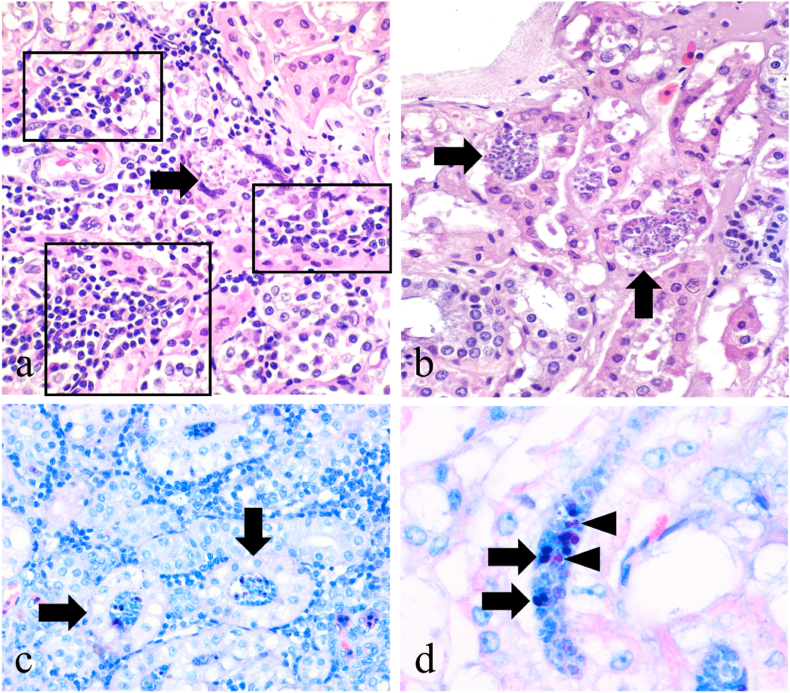
**Histomorphological characterisation of myxosporidiosis in renal tissue of *Emydura macquarii* - Turtle 6**. **a)** Chronic moderate multifocal to coalescing inflammatory infiltrates represented by lymphocytes, plasma cells and macrophages are infiltrating and expanding the renal cortical interstitium (black squares and rectangles), surrounding tubules containing myxozoan parasites (arrow). Haematoxylin and eosin (HE), 40X. **b)** Myxozoan parasites were observed mostly within tubular epithelial cells, represented by sporogonic plasmodia and spores; the latter measuring about 10um long x 5um wide (arrow). HE, 40X. **c)** Spore stages were also observed filling the lumen of intact proximal tubules (arrows), although interstitial renal inflammation was present in all cases when parasites were visible. Giemsa stain, 40X. **d)** Mature spores have an oval body, enclosing two polar pyriform capsules staining deep blue-purple (arrows) and one or two visible nuclei, staining magenta (arrowheads). Giemsa stain, 60X.

Gross examination was unremarkable in most organ systems, except mild kyphosis of the carapace at the level of the 4th and 5th vertebral scutes. Renal histopathology revealed a chronic tubulointerstitial nephritis (Fig. 1a) with intratubular myxozoan parasites in various stages of development (Fig. 1b), causing tubular degeneration (Fig. 1c) and partial to complete necrosis of the epithelium with mild hyperplasia, indicating a regenerative response. Parasite structure was best visualised on light microscopy on high magnification (40X, 60X) and with the Giemsa stain, which highlighted some of the principal ultrastructural components (Fig. 1d). Faecal flotation testing returned negative for any parasite or parasite eggs. The biochemical profile was unremarkable apart from a mild hypoglycaemia (2.4 mmol/L; reference range: 3.5–8.4 mmol/L) and mild hyperglobulinaemia (26 g/L; reference range: 0–23 g/L). All other parameters were within established reference intervals for reptiles.

#### 3.1.2 Turtle 11

As with Turtle 6, in Turtle 11 gross examination of organs was unremarkable, except for mild kyphosis of the carapace at the level of the 2nd and 3rd vertebral scutes. On renal histopathology, there was a mild chronic tubulointerstitial nephritis associated with the presence of myxozoan organisms in various stages of development within proximal tubules, associated with tubular degeneration and multinucleated giant cells. Faecal flotation testing returned negative for parasites or parasite eggs. The biochemical profile was unremarkable apart from a moderate hypoglycaemia (0.4 mmol/L; reference range: 3.5–8.4 mmol/L), mild hypoalbuminemia (9 g/L; reference range: 12–22 g/L) and mild decrease in AST (52 U/L; reference range: 54–181 U/L). All other parameters were within established reference intervals for reptiles.

#### 3.1.3 Turtle 13

Similar to Turtle 6 and 11, gross examination of most organ systems in Turtle 13 was unremarkable. Renal histopathology indicated a chronic tubulointerstitial nephritis associated with areas in which cortical tubules contained myxozoan organisms in various stages of development. Faecal flotation testing returned negative for parasites or parasite eggs. The biochemical profile was unremarkable apart from a moderate hypoglycaemia (1.3 mmol/L; reference range: 3.5–8.4 mmol/L), mild hypophosphatemia (0.9 mmol/L; reference range: 1.1–2.8 mmol/L), mild hypoalbuminemia (10 g/L; reference range: 12–22 g/L), mild hyperglobulinaemia (24 g/L; reference range: 0–23 g/L) and mildly elevated CK (1687 U/L; reference range: 100–1360 U/L). All other parameters were within established reference intervals for reptiles.

### 3.2 Molecular analysis

Four samples out of 13 were positive on PCR for myxozoa (Table 2), with sequences successfully obtained from 3 samples. Contigs assembled from overlapping sequences were between 1741 and 1757 base pairs in length, and were largely identical except for two positions, which varied between C, T and Y bases across the three contigs. Ambiguous bases were observed in the same positions in both forward and reverse reads in all three samples suggesting the presence of a mixture of at least two genotypes. Ambiguous bases or mismatches with low quality that occurred early or late in the contigs with incomplete overlap between sequences were removed. Sequences were deposited in GenBank under accession numbers PQ586236 – PQ586238. After alignment with reference GenBank sequences and trimming, a total of 1250 positions were available for the phylogenetic analysis.

**Table 2:** Turtle samples positive for renal inflammation and myxozoan infection under histological examination, PCR and sequencing.

Sample ID	Renal Inflammation	Myxozoa on Histopathology	PCR	Sequencing
Turtle 1	**✓**	**✗**	**✓**	**✓**
Turtle 2	**✗**	**✗**	**✗**	**✗**
Turtle 3	**✗**	**✗**	**✗**	**✗**
Turtle 4	**✗**	**✗**	**✗**	**✗**
Turtle 5	**✓**	**✗**	**✗**	**✗**
Turtle 6	**✓**	**✓**	**✓**	**✗**
Turtle 7	**✗**	**✗**	**✓**	**✓**
Turtle 8	**✗**	**✗**	**✗**	**✗**
Turtle 9	**✗**	**✗**	Not Run	Not Run
Turtle 10	**✓**	**✗**	**✗**	**✗**
Turtle 11	**✓**	**✓**	Not Run	Not Run
Turtle 12	**✓**	**✗**	**✓**	**✓**
Turtle 13	**✓**	**✓**	Not Run	Not Run

**Fig. 2:**
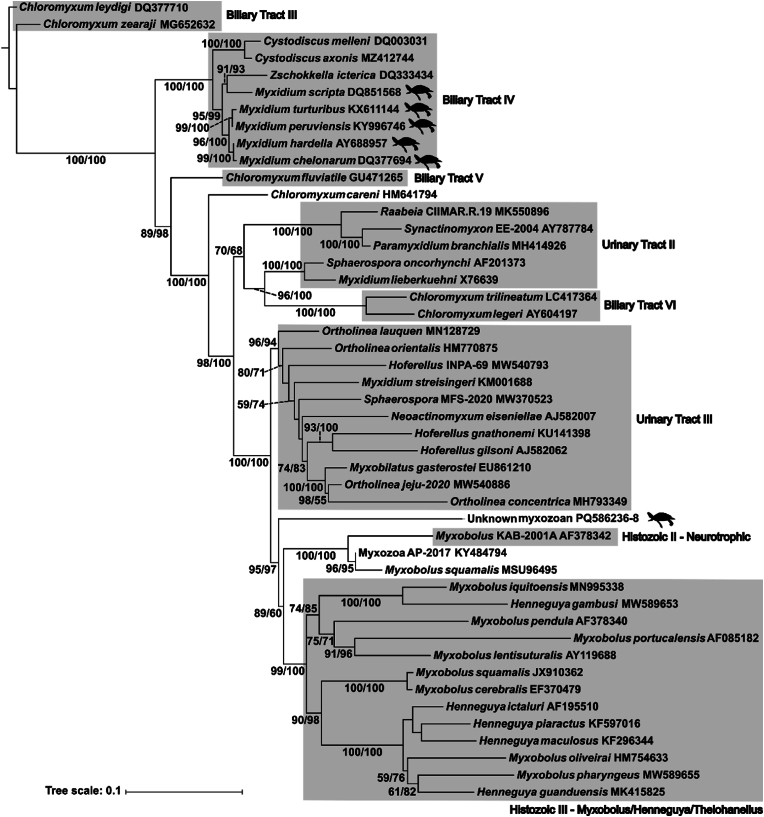
Maximum likelihood tree showing relationships between the unknown myxozoan (*Bivalvulida* sp.) and other taxa based on 18S rDNA sequences. Scale bar indicates the number of substitutions per site. Branch support values are comprised of UltraFast bootstrap values/posterior probabilities from corresponding Bayesian Inference tree; branch support is only shown where one or both values were greater than 70 %. Clades are identified with reference to Holzer et al. (2018). Icons indicate taxa reported from turtle species.

The Maximum Likelihood and Bayesian Inference analyses produced trees with very similar topology. Phylogenetic analysis of 18S sequences positioned the unknown myxozoan ex. *E. macquarii* in its own clade sister to Histozoic clades II (Neurotrophic) and III (Myxobolus/Henneguya/Thelohanellus) (Fig. 2) as identified by Holzer et al. (2018). These clades largely contain parasites of teleosts and polychaetes. Clades containing myxozoa infecting the host urinary tract (Urinary Tract clades II and III), biliary tract (Biliary Tract V and VI) and those infecting other turtle species (*Myxidium* spp.) occur basally to the unknown parasite.

Although four samples tested positive with PCR with myxozoan primers, and renal inflammation was observed in parasite-negative samples, only three DNA sequences were suitable for molecular analysis (Table 2). In all cases the DNA extraction control was negative.

The 18S rDNA sequences were compared with those deposited on the National Centre for Biotechnology Information (NCBI) Genbank database. The closest matches are presented in Table 3 and included *Myxobolus* sp. (MG250286) (94.3 % similarity) isolated from Bluntnose knifefish (*Brachyhypopomus beebei*) in Brazil. The next most closely related species were *Ortholinea* spp. with <94.1 % similarity isolated from fish.

**Table 3:** Closest NCBI Genbank matches for *Myxozoa* 18S rDNA sequences.

Sequence ID	Closest Genbank Match (Accession No.)	Percent Similarity	Query Cover	Host	Location	Publications
Turtle 1 (1754bp)	*Myxobolus* sp.MG250286)	94.3 %	55 %	Bluntnose knifefish (*Brachyhypopomus beebei)*	Brazil	Sindeaux-Neto et al., 2021
	*Ortholinea argusi* (MH197371)	94.1 %	64 %	Spotted scat (*Scatophagus argus*)	Malaysia	Unpublished
Turtle 7 (1757 bp)	*Myxobolus* sp.MG250286)	95.3 %	66 %	Bluntnose knifefish (*B*. *beebei)*	Brazil	Sindeaux-Neto et al., 2021
	*Ortholinea argusi* (MH197371)	94.1 %	64 %	Spotted scat (*S*. *argus*)	Malaysia	Unpublished
Turtle 12 (1741 bp)	*Myxobolus* sp.MG250286)	94.3 %	67 %	Bluntnose knifefish (*B*. *beebei)*	Brazil	Sindeaux-Neto et al., 2021
	*Ortholinea argusi* (MH197371)	94.1 %	67 %	Spotted scat (*S*. *argus*)	Malaysia	Unpublished

The phylogenetic reconstruction (Fig. 2) showed that the unknown myxozoan was distinct from other *Myxobolus* species and formed its own clade with strong support.

## 4 Discussion

This case series focuses on the histopathology and phylogenetic characterisation of an unknown myxozoa parasite discovered within the kidneys of *E. macquarii*, associated with significant chronic inflammation. This finding is comparable to a previous report illustrating lesions caused by *Myxidium hardella* in 2 Crown River turtles (*Hardella thurjii*) in Pakistan (Garner et al., 2005). Both turtles presented severely emaciated. Similar to our study, renal lesions were observed within the infected Crown River turtles including tubular necrosis and mineralization, chronic interstitial nephritis, membranoproliferative and mesangioproliferative glomerulopathy. Both Crown River turtles were diagnosed with chronic renal insufficiency secondary to myxozoanosis and subsequent metastatic mineralization. One turtle died due to severe health complications while the other was euthanised on welfare grounds (Garner et al., 2005).

The immune response to myxozoan infections, particularly in the kidneys, is complex and influenced by the parasite's invasion mechanisms. In general, myxozoans can invade immunoprivileged sites, including the central nervous system (CNS), eyes, and gonads, which may allow them to evade initial immune detection (Ferguson et al., 2008, Hemananda et al., 2009). If the parasite is not controlled early in the infection, it can reach these protected areas, where immune surveillance is limited (Gómez et al., 2014). The presence of inflammatory cells in the affected renal tissue indicates a robust immune response, characteristic of histozoic parasitic infections (Gómez et al., 2014). Such responses highlight the potential virulence of this species, as the inflammatory process can lead to renal dysfunction. The absence of the parasite and inflammation from CNS, eyes, and gonads, indicates this parasite does not appear to exploit immuno-privileged tissues. Instead, it seems to trigger a significant immune reaction upon invasion of the kidneys, similar to responses documented in other myxozoans affecting various fish species (Alvarez-Pellitero et al., 2008).

The renal lesions observed correlate with immune-mediated damage as the host attempts to combat the infection. Studies have shown that myxozoans can induce specific cellular immune responses in the kidneys (Cuesta et al., 2006, Sitjà-Bobadilla et al., 2006). For example, increased renal leukocyte infiltration, similar to what was observed in this study, and inflammatory markers, have also been observed in fish infected with myxozoans, suggesting an active immune engagement in response to the parasitic invasion (Sitjà-Bobadilla, 2008). In turbot infected with *Enteromyxum scophthalmi*, for instance, leukocyte numbers and respiratory burst activity increased in the kidneys, indicating a heightened immune response to the pathogen (Sitjà-Bobadilla et al., 2006). The regulation of cytokines such as IL-1β and TNFα has been documented in the context of myxozoan infections (Sitjà-Bobadilla, 2008). In the kidneys of affected turtles, similar patterns may emerge, with pro-inflammatory cytokines potentially driving the inflammatory response observed in the renal tubules. Consistent with an active immune response, mild hyperglobulinaemia was observed in 2/3 of the affected turtles. This elevation of globulins may reflect chronic stimulation secondary to persistent myxozoan infection. Given the presence of renal lesions indicative of inflammation, an immune-mediated origin is plausible. However, the absence of other significant biochemical abnormalities limits conclusions on the systemic impact of this infection.

Every turtle enlisted into the study were in good body condition, and no notable associations were found between PFAS levels, altered lipidomics, metabolomics and proteomics and myxozoan infections (Beale et al., 2024). The presence of lymphoplasmacytic infiltrates within the renal interstitium and associated tubular degeneration suggests an active host immune response, indicative of a pathogenic process. However, renal tubular degeneration is a non-specific finding in free-ranging reptiles and can occur secondary to various aetiologies, including bacterial infections, environmental stressors, severe dehydration, and dietary factors. While other secondary causes of renal degeneration cannot be ruled out, the degree of inflammation and tissue changes suggests that this myxozoan species has the potential to contribute to renal dysfunction. The turtles in the study appeared to be in adequate nutritional condition and hydration with no clinical signs recorded prior to euthanasia for the PFAS study, limiting conclusions on the broader health impact of this parasitic infection. Nonetheless, given the known pathogenic potential of some myxozoans and the renal changes observed, further investigations are warranted to determine whether co-factors such as environmental contaminants or host susceptibility influence disease severity.

The molecular analysis supports that this myxozoan parasite might represent an unknown species, most similar to *Myxobolus* sp. (94.3–95.3 % similarity) and *Ortholinea argusi* (94.1 % similarity) (Table 3). However, assignment to the genus level remains uncertain. The 18S sequence similarity only differed by 0.2 % between these species, but given the known polyphyletic and paraphyletic nature of these genera, molecular data alone are insufficient for taxonomic resolution. Additionally, DNA sequences are available for only a fraction of nominal species. As such, genus-level classification in myxozoans relies heavily on myxospore morphology, which is unavailable in this study. Nonetheless, the sequence data support its placement within the order Bivalvulida. Further morphological and ultrastructural analyses are required to determine its precise taxonomic position.

The discovery of this myxozoan species represents an advancement in our understanding of myxozoan diversity, marking the first published incidence of a myxozoan parasite in turtles in Australia as well as the first molecularly confirmed incidence of myxozoa in Murray River turtles. These findings are particularly noteworthy not only for their geographic novelty but also for their pathogenic impact, as it induces renal lesions of at least moderate severity in its Murray River turtle hosts. Unlike many other myxozoans that exhibit relatively benign effects, this species may pose a health impact to affected turtles. Although epithelial hyperplasia – an indicator of regenerative response – was observed in the kidneys of infected turtles, blood biochemistry findings, including a mild hyperglobulinaemia suggests a possible systemic immune response. However, additional clinical pathology data, including electrolytes evaluation, creatinine, packed cell volume, leukocyte counts would be necessary to fully elucidate the extent of any renal dysfunction and its broader physiological impact.

The ecological and epidemiological implications of this discovery are yet to be fully elucidated, necessitating further studies to determine whether the presence of this parasite in Murray River turtles reflects a recent introduction into the host or the ecosystem. Such an introduction could alter population dynamics and affect interactions with other infectious agents. Interestingly, despite the presence of this pathogen, all infected turtles exhibited adequate body condition and were found to have shelled oviductal eggs. Typically, optimal health is a recognised pre-requisite for successful reproduction (Alfaro et al., 2008); however, these findings suggest that reproductive capacity may persist in *E. macquarii* even in individuals facing health challenges.

Previous reports of myxozoan infections in multiple species of native Australian frogs (Hartigan et al., 2013) highlight the necessity for increased awareness and monitoring of these pathogens within aquatic ecosystems. *Cystodiscus australis* and *Cystodiscus axonis*, have been shown to infect Australian frogs, causing significant harm due to their ability to induce liver and neurological damage in infected tadpoles. Although not all host species are affected the same way, both *Cystodiscus* species cause similar inflammatory and hyperplastic lesions in the livers of infected frogs. Contrastingly, the brain lesions caused by *C. axonis* are more severe than those seen in *C. australis* infections in some host species (Hartigan et al., 2013). Additionally, *Myxobolus* infections have been reported in multiple species of native Australian frogs, including the endangered *Litorea aurea* and *Litoria raniformis*, although only as incidental findings. Specifically, *Myxobolus falla*x and *Myxobolus hylae* have been identified in the gonads of Australian frogs with infected individuals showing signs of lethargy and diminished fertility, possibly impacting reproductive success (Hartigan et al., 2013).

Conservation concerns are heightened regarding the impact of this parasite on *E. macquarii* and other endangered turtle species inhabiting ponds in South-East Qld. Populations of *E. macquarii* are already experiencing significant declines, with studies indicating a reduction of 69–91 % between 1976 and 2011 (Chessman, 2011), resulting in its classification as Threatened in the state of Victoria, Australia. Many other Australian freshwater turtle species are also facing similar important declines (Chessman, 2011, Van Dyke et al., 2018). Evidence of renal damage suggests this unknown myxozoan may affect host health, warranting further investigation into its ecological significance.

The potential health impacts of this myxozoan species could extend beyond turtles, affecting a broader range of aquatic organisms and potentially disrupting local ecosystems. Despite the significance of this finding and the prominence of the fish farming industry in the country, our knowledge of Australian myxozoans remains limited. Further investigations are warranted to understand the life cycle, risk of transmission to farmed fish and other aquatic animals, as well as its potential for zoonotic or interspecies transmission. Additionally, subsequent studies would assist in clarifying its conservation implications and support the development of strategies for managing the health of affected populations, ensuring that interventions are informed by a thorough understanding of the ecological dynamics at play.

### 4.1 Limitations

PCR analysis identified the presence of myxozoan DNA in four turtles, whereas histological examination revealed evidence of the parasite in only three. This discrepancy may be attributed to the inherent limitations of histological sampling, where the specific tissue sections examined may not have contained the parasite, despite its presence in other tissue areas. Consequently, the positive PCR results likely reflect the true distribution of the parasite, missed in histological sections. Further histological sampling to enhance detection was not feasible due to funding constraints.

The remaining 4 turtles, although showing renal tubular degeneration, did not have detectable myxozoan infections on histological examination. Possible explanations include: sparse distribution of the parasite in renal tissue, cleared myxozoan infection but unresolved renal inflammation, or renal inflammation induced by factors unrelated to the myxozoan infections – other pathogens, environmental stressors or intrinsic renal disorders (Zwart, 2006). Further characterisation using electron microscopy (EM) was attempted; however, the parasite was not observed in the sections analysed. Due to funding constraints, additional imaging to investigate the ultrastructure and other morphological features of the parasite could not be pursued.

Biochemical analysis was conducted using a reptile-specific panel; however, it is important to note that these tests have not been specifically validated for Murray River turtles. As such, the reference ranges used for interpretation may not accurately reflect species-specific physiological parameters and should be interpreted with caution when assessing the clinical significance of biochemical abnormalities.

## 5 Conclusion

We have identified an unknown myxozoan parasite with a phylogenetic positioning in a distinct clade emphasising its uniqueness within its Myxozoa group in Murray River turtles. This finding represents an advancement in the field of parasitology and wildlife conservation in Australia. The association of this parasite with renal lesions underscores its pathogenic potential, distinguishing it from many other myxozoans that typically elicit minor tissue damage. Given the concern for potential impact on turtle health, this discovery raises conservation concerns, particularly regarding the potential long-term effects on Murray River turtle populations.

### 5.1 Future research directions

Further research should prioritise several key areas to thoroughly investigate the biological and ecological implications of this unknown myxozoan species, particularly concerning freshwater turtles experiencing urinary inflammation or lesions in other organ systems. Detailed studies on host specificity are required in other aquatic and semi-aquatic hosts. Moreover, elucidating its developmental stages, transmission routes, and environmental factors that influence its life cycle will facilitate the development of effective management and control strategies. Future studies should also employ EM and morphological analysis of spores, further supporting characterisation and taxonomy.

Comprehensive surveys are necessary to assess the prevalence of this parasite and spread across different turtle populations and regions in Australia. Such surveys should include investigations into ecosystem conditions, intermediate hosts, co-existence with both hosts and other pathogens to better evaluate its impact on turtle health and transmission at a broader scale. Further research is essential to understand the ecological and epidemiological implications of this parasite, as well as to inform conservation strategies aimed at mitigating its impact on vulnerable turtle populations.

## CRediT authorship contribution statement

**Zachary Low:** Writing – review & editing, Writing – original draft, Visualization, Investigation, Data curation. **Telleasha L. Greay:** Writing – review & editing, Methodology, Investigation, Formal analysis. **Swaid Abdullah:** Writing – review & editing, Supervision, Resources, Methodology, Investigation, Funding acquisition. **Phoebe A. Chapman:** Writing – review & editing, Visualization, Resources, Methodology, Investigation, Formal analysis, Conceptualization. **Viviana Gonzalez-Astudillo:** Writing – review & editing, Supervision, Project administration, Methodology, Investigation, Funding acquisition, Formal analysis, Data curation, Conceptualization.

## Ethics statement

*Emydura macquarii* were obtained opportunistically from the secondary use of carcasses obtained for another study conducted as per the Queensland Department of Environment and Science (DES) Wildlife and Threatened Species animal population dynamics survey (AEC permit SA, 2018/11/663).

## Funding

No funding was obtained for this project.

## Declaration of competing interests

The authors declare that they have no conflict of interest regarding the publication of this manuscript. The research was conducted in the absence of any commercial or financial relationships that could be construed as a potential conflict of interest.
